# Clinical assessment of patient outcomes post percutaneous pulmonary valve implantation: Insights from a single tertiary centre

**DOI:** 10.21542/gcsp.2024.47

**Published:** 2024-11-01

**Authors:** Sushant Saluja, Daniel Myers, Bernard D. Keavney, Freidoon Keshavarzi, Simon G. Anderson

**Affiliations:** 1 Division of Cardiovascular Sciences, Faculty of Biology, Medicine and Health, The University of Manchester, UK; 2 Division of Medicine and Manchester Academic Health Science Centre, Manchester University NHS Foundation Trust Manchester, Manchester, UK; 3 Caribbean Institute for Health Research, The University of the West Indies, Mona, Jamaica

## Abstract

Background: Percutaneous pulmonary valve implantation (PPVI) has emerged as a promising treatment for congenital right ventricular outflow tract (RVOT) dysfunction and restoring conduit graft viability.

Methods: This is a single-centre retrospective study of 41 patients (18 men, 23 women; mean age 26.1 ± 10.2 years) who underwent PPVI between December 2007 and November 2014 and were evaluated for right ventricular pressures and exercise tolerance.

Results: PPVI significantly reduced mean baseline RVOT gradients across different pathologies: stenosis (45 *vs* 18.4 mmHg), regurgitation (19.2 *vs* 7.6 mmHg), and mixed disease (32.5 *vs* 12 mmHg). Furthermore, mean right ventricular (RV) systolic pressures decreased from 61.6 ± 2.3 to 41.9 ± 2 mmHg (*p* < 0.001), while RV diastolic pressures decreased by about 60% from 14.3 ± 1.1 to 8.6 ± 1.4 mmHg (*p* < 0.001). Echocardiography revealed significant improvements in pulmonary and tricuspid valve velocities (*p* for trend < 0.01). Additionally, there was a consistent reduction in the main pulmonary artery maximum pressure gradient measured pre-procedure. No significant changes were observed in PR, QRS, or QTc interval duration on follow-up electrocardiograms. Similarly, no changes were noted in cardiopulmonary exercise test performance during follow-up.

Conclusion: The study highlights the effectiveness of PPVI using Medtronic Melody and Edwards SAPIEN valves in patients with various pulmonary diseases. Immediate improvements in right ventricular pressures and functional outcomes suggest that PPVI is a valuable treatment option for individuals with RVOT dysfunction. Multi-centre collaborations are crucial for further elucidating the long-term effects of PPVI on cardiac function, exercise tolerance, and quality of life in RVOT dysfunction.

## Introduction

Congenital or acquired heart conditions involving dysfunction of the right ventricular outflow tract (RVOT) necessitate the implantation of prosthetic valves, with or without conduits. However, recurrent surgical interventions due to the deterioration of prosthetic valves and conduits results in increased scarring, heightened surgical risks, psychological strain, and prolonged recovery fperiods for patients undergoing open-heart surgery^1^. Percutaneous pulmonary valve implantation (PPVI) with Melody and Edwards SAPIEN valves offers a non-surgical option for pulmonary valve replacement, reducing the need for multiple surgeries and extending the lifespan of RVOT revisions^2,3^. Widely recognized as the primary approach in selected patients, PPVI utilizes the Melody valve, a bovine jugular venous valve within a platinum-iridium stent, or the Sapien XT valve, a bovine pericardial valve in a cobalt-chromium stent. Since its introduction in 2000 and FDA approval in 2010, PPVI has gained global acceptance, replacing surgically implanted right ventricle to pulmonary artery (RV-to-PA) valves and conduits^4,5^.

Despite these advancements, data discrepancies from limited studies examining changes in intracardiac pressures, echocardiograms, cardiopulmonary exercise testing, and electrocardiograms post-PPVI. A comprehensive understanding of right ventricular function post-procedure is imperative. This study aims to evaluate the early and late procedural and clinical outcomes of PPVI utilising both Melody and Edwards SAPIEN valves at a single tertiary centre.

## Methods

### Study population

This retrospective, non-randomized study was undertaken at the Manchester Heart Centre in the United Kingdom to evaluate patients who exhibited dysfunction in their right ventricular outflow tract (RVOT) conduits and subsequently underwent percutaneous pulmonary valve implantation (PPVI) utilising either a Melody or Edwards-SAPIEN valve.

The determination of valve selection was influenced by specific anatomical and procedural considerations unique to each patient. The Melody valve was predominantly selected for patients with narrower RVOT conduits, particularly those measuring up to 22 mm in diameter; its enhanced flexibility and adaptability to irregular anatomies facilitated optimal implantation. This valve proved especially beneficial for patients with prior conduit repairs or with calcified, rigid structures, where the inherent flexibility of the valve was critical for navigating the complex anatomical landscape.

Conversely, the Edwards-SAPIEN valve, a balloon-expandable, bovine pericardial valve, was favoured in scenarios involving larger RVOT conduits or native outflow tracts exceeding 23 mm in diameter^6^. In such cases, the rigid framework of the valve offered superior support and long-term stability within the larger annulus^6^. The selection of this valve type was mainly aimed at patients with more rigid structures, ensuring enhanced anchoring and minimising the risk of valve migration or malposition.

The process for valve selection was anchored in comprehensive pre-procedural assessments, including echocardiography, cardiac MRI, and angiography, which allowed for precise measurements of the conduit or native outflow tract diameter and the identification of any calcification or prior surgical interventions. The study encompassed the period from December 2007, when the procedure was initially introduced, to November 2014; patients treated beyond this timeframe were excluded due to limitations in follow-up data availability beyond an adequate observational period.

Criteria for PPVI included pulmonary stenosis in the right ventricular outflow tract, characterised by an RVOT pullback gradient and RV pressure > 25 mmHg and 2/3 systemic pressure, respectively. Patients with an RVOT pressure < 25 mmHg were classified as having pulmonary regurgitation, while those not meeting either criterion were categorised as having a mixed lesion^7^. Clinical features, such as exercise intolerance, heart failure symptoms, ECG abnormalities, were also considered. Suitability for Melody or Edward SAPIEN valve implantation involved thorough evaluation *via* transthoracic echocardiography (TTE), cardiac computed tomography (CT), cardiac magnetic resonance imaging(MRI), and cardiac angiography to assess anatomy and feasibility.

### Clinical assessment

Regular follow-up evaluations were performed at 1, 6, and 12-month intervals until June 2015 (data accessible within our centre up to that point), focusing on assessing the feasibility and safety of valve implantation. We comprehensively evaluated patient responses using physical examinations, exercise tests, and transthoracic echocardiography. Hemodynamic parameters, such as RV and PA systolic pressures, were assessed *via* cardiac catheterisation and quantifying the peak systolic gradient across the right ventricular outflow tract (RVOT) using Doppler recordings on echocardiography. Pulmonary regurgitation (PR) and tricuspid regurgitation (TR) were delineated *via* transthoracic echocardiography. Additional measures of functional capacity, including peak oxygen consumption (VO2), and metabolic equivalents (METS), were also measured. Adverse events, including death, reinterventions, infective endocarditis, and melody stent fractures, were monitored post-percutaneous pulmonary valve implantation, with those occurring after one month considered delayed complications.

### Procedural data

All procedures were conducted under general anaesthesia, with or without endotracheal intubation and ventilation through a femoral approach. Implantation adhered to established protocols involving preprocedural hemodynamic assessment of right atrial, right ventricular (RV), pulmonary artery (PA), and aortic (Ao) pressures. Balloon interrogation of the right ventricular outflow tract (RVOT) was conducted concurrently with aortic root or selective coronary artery angiograms to assess valve landing zones. Deployment of Melody or Edwards-SAPIEN valves depended on conduit diameter, utilising respective transcatheter delivery systems. Post-deployment angiography verified optimal valve function, with additional balloon dilatations performed if needed. Haemostasis was ensured using a Perclose device (https://www.cardiovascular.abbott/us/en/hcp/products/peripheral-intervention/vessel-closure/perclose-proglide-suture-mediated-closure-system/single-device-deployment.html) for venous closure and an Angioseal device for arterial closure (https://www.terumois.com/products/closure/angio-seal-vascular-closure-devices/angio-seal.html). Patients received intravenous Heparin (5,000 units) and antibiotics, typically IV Augmentin and Gentamicin, and were prescribed lifelong Aspirin therapy (75 mg once daily), unless contraindicated.

### Statistical analysis

The cohort was organised into two groups for comparative analysis of key clinical parameters before and after pulmonary valve implantation, with follow-up assessments conducted at 1 month, 6 months, 12 months, and at the most recent visit in June 2015. Given the repeated measurements obtained from the same subjects at these multiple time points, repeated-measures ANOVA was selected to evaluate longitudinal changes. This method was chosen for its ability to detect within-subject differences across several time points, which is essential for accurately assessing temporal changes in continuous variables. Repeated-measures ANOVA effectively manages within-subject comparisons and accounts for data correlation across uniform time intervals, enhancing sensitivity by reducing between-subject variability^8^.

While paired t-tests could theoretically be used to compare each time point (pre- *vs* each follow-up), they are not ideal when multiple groups are involved and would result in a higher risk of Type I error due to repeated testing^9^. Repeated-measures ANOVA allows us to analyse all time points in a single model, providing a more comprehensive view of changes over time without inflating error rates. Categorical variables were analysed using the Chi-squared test, while continuous variables were assessed with Student’s *t*-test or one-way ANOVA if parametric assumptions held^10^. If these assumptions had not been met, we would have employed a non-parametric alternative, such as the Wilcoxon signed-rank test^11^. Continuous variables were presented as means with standard errors (SE) or 95% confidence intervals (CIs), while categorical variables were reported as counts (n) and percentages (%). For ANOVA analyses, F-statistics and associated *P*-values were reported to provide insights into effect size and statistical significance (with *P* < 0.05 considered significant). Confidence intervals were also offered for critical outcomes to clarify the range and clinical relevance of observed changes. All analyses were conducted using Excel and Stata/MP version 13.1, and Python 3.3.1.

**Table 1:** Overview of patient demographics, primary indications for percutaneous pulmonary valve implantation (PPVI), and valve implant statistics.

**Clinical characteristics**	**Baseline**
**Age at procedure years (SE)**	26.1 (22.9–29.3)
**Gender M *vs* F n (%)**	23 *vs* 18 (56 *vs* 44%)
**Primary indication for PPVI n (%)**	
Pulmonary atresia	10 (24)
Ross procedure	9 (22)
TGA	4 (10)
TOF	15 (37)
Truncus arteriosus	3 (7)
**Primary Indication for procedure n (%)**	
Pulmonary stenosis	19 (54)
Pulmonary regurgitation	10 (29)
Mixed pulmonary valve disease	6 (17)
**Transcatheter intervention device used n (%)**	
Melody	32 (78)
Edwards SAPIEN	5 (12)
Missing data	4 (10)
Duration of procedure (minutes)	207
**Valve size n (%)**	
Melody 18 mm	5 (16.7)
Melody 20 mm	17 (53.3)
Melody 22 mm	9 (30)
Edwards SAPIEN 23 mm	4 (80)
Edwards SAPIEN 26 mm	1 (20)
**NYHA Class n (%)**	
I	19 (61.3)
II	8 (25.8)
III	4 (13)
**Mean LV ejection fraction %**	66
**Mean follow-up time Years (SDEV)**	3 (1.96)

**Notes.**

Abbreviations SE Standard Error M Male F Female n Number PPVI Percutaneous Pulmonary Valve Implantation TGA Transposition of the Great Arteries TOF Tetralogy of Fallot LV Left Ventricle NYHA New York Heart Association SDEV Standard Deviation

## Results

**Figure 1.**
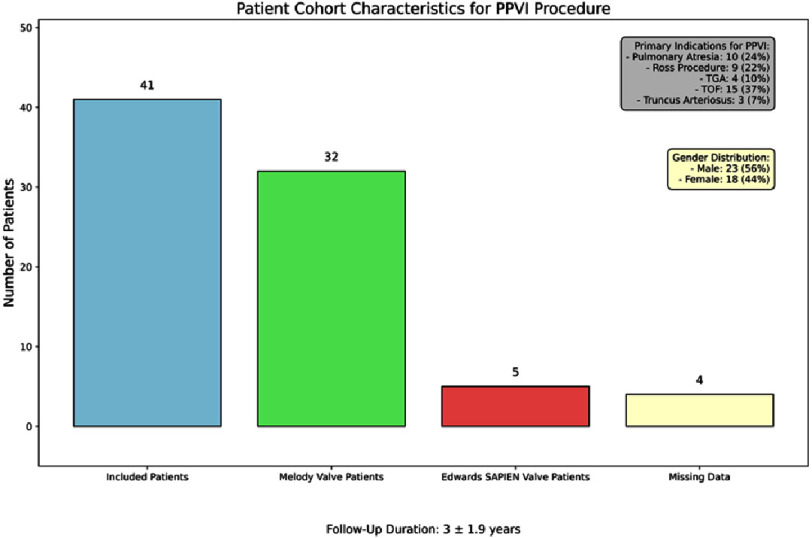
Clinical characteristics of a cohort of 41 patients who underwent Percutaneous Pulmonary Valve Implantation (PPVI). Among the patients, 78% (*n* = 32) received the Melody valve, while 12% (*n* = 5) received the Edwards SAPIEN valve, with 10% (*n* = 4) missing data on valve type. Primary indications for PPVI included pulmonary atresia (24%), prior Ross procedure (22%), transposition of the great arteries (TGA) (10%), Tetralogy of Fallot (TOF) (37%), and truncus arteriosus (7%). For the procedures, the primary indications were pulmonary stenosis (54%), pulmonary regurgitation (29%), and mixed pulmonary valve disease (17%). The cohort comprised 56% male (*n* = 23) and 44% female (*n* = 18) patients. Follow-up duration averaged 3 years (±1.9 years), reflecting patient outcomes post-PPVI.

Table 1 and Figure 1 outline the demographic and clinical characteristics of the 41 patients, with 56% male and 44% female participants. Common cardiac diagnoses included Tetralogy of Fallot (*n* = 15), Pulmonary Atresia (*n* = 10), Ross procedure (*n* = 9), Transposition of the Great Arteries (*n* = 4), and Truncus Arteriosus (*n* = 3). Notably, 44% of patients had pulmonary stenosis, while pulmonary regurgitation and mixed pulmonary valve disease affected 34% and 22% of patients, respectively. The mean follow-up duration was 3 ± 1.9 years, ranging from 6 months to 7 years. The Melody valve was predominant, used in 78% of cases, compared to the Edwards SAPIEN valve in 12% of cases. Among Melody valve recipients, the most common sizes were 20 mm (53.3%), 22 mm (30%), and 18 mm (16.7%). Conversely, the 23 mm Edwards SAPIEN valve was predominantly used (80%), with only one instance of the 26 mm variant (20%). Most patients had good functional capacity at baseline (NYHA class I), with a mean left ventricular ejection fraction of 66%.

### Intracardiac pressures

Table 2 summarizes invasive pressure data from cardiac catheterization. Post- implantation, right ventricular systolic pressures dropped by 30% (61.6 to 41.9 mmHg, *F*= 31.6; *P* < 0.001), and the pulmonary artery-right ventricular pullback gradient decreased by nearly 60% from 35.9 to 15 mmHg (*P* < 0.001). Additionally, there was a significant decrease in right ventricular diastolic pressures (*F* = 9.9; *P* = 0.003). While pulmonary artery and aortic diastolic pressures remained unchanged post-implantation, aortic systolic pressures increased from 99 to 108 mmHg (*P* = 0.03). Stratification by primary indication for the procedure revealed an average 60% reduction in right ventricular outflow tract gradient overall (45 mmHg to 18.4 mmHg in stenosis, 19.2 mmHg to 7.6 mmHg for regurgitation and 32.5 mmHg to 12 mmHg for mixed disease; *P* for trend < 0.001). Furthermore, the ratio of right ventricular to aortic pressure decreased from 0.65 to 0.40 mmHg (*P* < 0.0001) between pre- and post-implantation, respectively.

### Echocardiographic data

Table 3 shows echocardiographic data. Mean pulmonary artery velocity significantly decreased from baseline (3.58 m/s) at one month (2.87 m/s), persisting at 1 year (2.88 m/s) and the most recent echo (2.83 m/s; overall *P* for trend < 0.0001). Maximum pulmonary artery pressure gradient (MPA max PG) also significantly reduced (*P* < 0.0001) (data available on request), consistent across baseline, one month, and the latest measurement (June 2015). This correlated with a decrease in tricuspid regurgitant gradient from baseline (59 ± 3 mmHg) to one month (42 ± 3 mmHg), maintained at 6 (50 ± 4 mmHg) and 12 months (43 ± 3 mmHg), and the most recent measurement (47 ± 4 mmHg) (*P* < 0.0013). Tricuspid regurgitant velocity (TR Vmax PG) also decreased significantly within one-month post-procedure (*P* < 0.0013). Right ventricular internal diameter in diastole (RVIDd) and tricuspid annular plane systolic excursion (TAPSE) remained unchanged. Overall, right ventricular function showed no significant changes over time, but a trend emerged with initially good function declining over time, coinciding with an increase in mild to moderate impairment.

**Table 2:** Internal cardiac catheter pressures before and after Percutaneous Pulmonary Valve Implantation (PPVI).

**Internal Cardiac Pressures (mmHg ± SE)**	**Baseline Mean (SE)**	**Baseline Confidence Interval**	**Post Procedure Mean (SE)**	**Post Procedure Confidence Interval**	***P* Value**
Right ventricular systolic pressure	61.6 (2.3)	[57.1, 66.1]	41.9 (2.7)	[36.6, 47.2]	*F* = 31.6; *P* < 0.001
Right ventricular diastolic pressure	14.3 (1.1)	[12.1, 16.5]	8.6 (1.4)	[5.9, 11.3]	*F* = 9.9; *P*= 0.003
Aortic systolic pressure	98.9 (2.5)	[94.0, 103.8]	108.4 (3.4)	[101.7, 115.1]	*F* = 5.1; *P*= 0.03
Aortic diastolic pressure	59.9 (3.1)	[53.8, 66.0]	66.4 (4.9)	[56.8, 76.0]	NS
Pulmonary artery systolic pressure	28.3 (1.7)	[25.0, 31.6]	29.5 (2.0)	[25.6, 33.4]	NS
Pulmonary artery diastolic pressure	11.9 (1.3)	[9.4, 14.4]	10.7 (1.8)	[7.2, 14.2]	NS
Pulmonary artery mean pressure	19.9 (1.4)	[17.2, 22.6]	24.3 (2.5)	[19.4, 29.2]	NS
PA-RV pull back gradient	35.9 (1.8)	[32.4, 39.4]	15.0 (1.8)	[11.5, 18.5]	*F*= 64.7; *P* < 0.001
RV/Aortic Pressure	0.65 (0.2)	[0.3, 1.0]	0.40 (0.15)	[0.1, 0.7]	*F* = 32.4; *P* < 0.0001

**Notes.**

Abbreviations SE Standard Error mmHg Millimeters of Mercury *F* *F*-value NS Not Significant PA Pulmonary Artery RV Right Ventricle

**Table 3:** Echocardiographic, ECG and CPET results by follow-up.

**ECHO Measurements**	**Baseline Mean (SE)**	**Baseline CI**	**One Month Mean (SE)**	**One Month CI**	**Six Months Mean (SE)**	**Six Months CI**	**One Year Mean (SE)**	**One Year CI**	**Most Recent Mean (SE)**	**Most Recent CI**
TAPSE cm	1.70 (0.07)	[1.56, 1.84]	1.68 (0.08)	[1.53, 1.83]	1.61 (0.08)	[1.46, 1.76]	1.75 (1.09)	[0.57, 2.93]	1.70 (0.09)	[1.52, 1.88]
RVIDd cm	4.4 (0.2)	[4.00, 4.80]	3.7 (0.2)	[3.30, 4.10]	4.2 (0.2)	[3.80, 4.60]	4.0 (0.3)	[3.40, 4.60]	4.2 (0.3)	[3.60, 4.80]
TR Vmax m/s	3.8 (0.1)	[3.59, 4.01]	3.2 (0.1)	[2.99, 3.41]	3.4 (0.1)	[3.19, 3.61]	3.2 (0.1)	[2.99, 3.41]	3.4 (0.1)	[3.19, 3.61]
TR Vmax PG mmHg	59 (3)	[53.5, 64.5]	42 (3)	[36.5, 47.5]	50 (4)	[46.0, 54.0]	43 (3)	[37.5, 48.5]	47 (4)	[43.0, 51.0]
MPA Vmax m/s	3.58 (0.11)	[3.37, 3.79]	2.87 (0.13)	[2.61, 3.13]	2.94 (0.13)	[2.68, 3.20]	2.88 (0.14)	[2.62, 3.14]	2.83 (0.14)	[2.57, 3.09]
MPA max PG mmHg	53 (3)	[47.5, 58.5]	35 (3)	[29.5, 40.5]	35 (3)	[29.5, 40.5]	34 (3)	[28.5, 39.5]	34 (3)	[28.5, 39.5]
PR interval [ms]	169.8 (8.8)	[152.5, 187.1]	177.8 (10.6)	[156.6, 199.0]	154.9 (12.4)	[129.8, 180.0]	178.6 (14.2)	[150.4, 206.8]	—	—
QRS [ms]	144.7 (6.0)	[132.9, 156.5]	145.4 (7.2)	[131.1, 159.7]	138.2 (8.9)	[121.5, 154.9]	149.0 (9.7)	[130.2, 167.8]	—	—
QTc [ms]	463.8 (7.5)	[448.9, 478.7]	475.2 (8.6)	[458.5, 491.9]	453.6 (10.1)	[433.7, 473.5]	468.0 (11.5)	[445.7, 490.3]	—	—
VO2% peak	63.2 (2.2)	[58.1, 68.3]	64.5 (3.3)	[58.3, 70.7]	—	—	—	—	—	—
VO2%@LT	42.4 (2.2)	[37.2, 47.6]	42.2 (2.9)	[36.5, 47.9]	—	—	—	—	—	—
METS	6.6 (0.43)	[5.75, 7.45]	5.4 (0.46)	[4.48, 6.32]	—	—	—	—	—	—
VECO2@LT	30.2 (0.95)	[28.3, 32.1]	30.7 (1.3)	[28.1, 33.3]	—	—	—	—	—	—
VECO2 slope	28.2 (1.0)	[26.2, 30.2]	27.5 (1.3)	[25.2, 29.8]	—	—	—	—	—	—

**Notes.** Certainly: Abbreviations – * June 2015.

Abbreviations NS not significant *P* < 0.05 significant TAPSE Annular Plane Systolic Excursion RVIDd Right Ventricular internal dimension in diastole TR Vmax Tricuspid regurgitant maximum jet velocity TR maxPG Tricuspid regurgitant maximum pressure gradient LVEF Left ventricular ejection fraction MPA Vmax Main pulmonary artery maximum jet velocity MPA maxPG main pulmonary artery maximum pressure gradient VO2%max percentage of predicted peak oxygen uptake VO2%@LT percentage of predicted oxygen uptake at anaerobic threshold METS Metabolic equivalent VECO2@LT pulmonary ventilation to carbon dioxide ratio at anaerobic threshold

The study found no ECG changes, including PR, QRS, and QTc durations, six months after percutaneous pulmonary valve implantation (PPVI) (Table 3). Similarly, cardiopulmonary exercise testing (CPET) showed no differences before and after valve implantation (Table 3). No significant adverse events were observed in the cohort, apart from one patient who developed endocarditis, which resolved with antibiotics.

## Discussion

This study conducted an analysis of data from 41 patients who underwent percutaneous pulmonary valve implantation (PPVI), demonstrating significant reductions in right ventricular pressures, improved valve function, and enhanced quality of life over a mean follow-up duration of 3 ± 1.9 years. These findings are consistent with previously documented immediate and long-term effects of PPVI, corroborating outcomes observed in prior studies^7,8^. However, the limited sample size (*n* = 41) constrains the statistical power and generalisability of the results, while the retrospective nature of data collection presents additional challenges. Moreover, variability in post-procedure assessments, particularly subjective echocardiographic measurements like right ventricular internal diameter in diastole (RVIDd), did not exhibit expected improvements despite the observed reductions in right ventricular pressures during diastole, further introducing potential variability. This study also identified discrepancies in outcomes relative to previous reports, notably concerning electrocardiographic parameters and cardiopulmonary exercise testing results^1,9,10^. Ongoing data collection at later time points would facilitate more profound insights into longitudinal trends and emphasise the necessity of continued research to capture these changes.

### Limitations of the current study

The principal limitations of this study pertain to its retrospective design and limited cohort size, which negatively influenced the statistical power and generalisability of the findings. These challenges were exacerbated by the presence of missing or inconsistent cardiac MRI data, which compromised the robustness of key variables. Moreover, variability in post-procedural assessments and subjective measurements, such as the right ventricular internal dimension in diastole (RVIDd) observed through echocardiograms, introduced potential inconsistencies.

The lack of a control group further constrained our ability to compare PPVI outcomes against alternative treatment options directly. Additionally, restructuring adult congenital heart disease (ACHD) services in North-West England, as initiated by NHS England in 2017, introduced further complexities. During this period, PPVI procedures were gradually centralised to Liverpool, while Manchester retained outpatient and low-complexity services, and hospitals such as Leeds, Newcastle, and Birmingham intermittently managed ACHD cases. This shift in care delivery led to fragmented records and inconsistent follow-up assessments due to the involvement of varying clinicians and protocols. Consequently, the quality and type of diagnostic data exhibited significant variability across different sites, complicating efforts toward standardisation.

Future investigations would benefit from a larger, prospectively collected cohort alongside standardised evaluation protocols, facilitating a more rigorous and comprehensive understanding of PPVI outcomes. Incorporating a control group and consistent post-procedural assessments, including 24-hour ECG monitoring and cardiac MRI, could enhance the validity of future findings, allowing for a more precise comparison of PPVI outcomes relative to alternative interventions. Despite these limitations, our study provides valuable insights into data collection practices within evolving healthcare systems and emphasises the critical necessity for a standardised approach in prospective ACHD research.

## Conclusions

This study accentuates the safety and efficacy of PPVI, utilising Melody and Edwards SAPIEN valves, among patients with pulmonary stenosis, regurgitation, or mixed pulmonary diseases, thereby positioning it as a viable alternative to open-heart surgery. The observed enhancements in right ventricular pressures and valve function are consistent with the procedure’s principal objectives of restoring cardiac function and improving patient quality of life. Nevertheless, further research is warranted to investigate the long-term repercussions of PPVI on cardiac function and the occurrence of arrhythmias post-procedure, potentially through the implementation of ambulatory ECG monitoring. Additionally, forthcoming studies involving more significant, prospectively compiled cohorts and standardised data collection methods will be essential to refining patient selection criteria and augmenting the applicability of PPVI in clinical settings.

## Funding

None.

## Competing interests

The authors declare that they have no competing interest.

## Author statements

Data Curation: Sushant Saluja, Danial Myers, and Simon G. Anderson

Formal Analysis: Sushant Saluja, Danial Myers, and Simon G. Anderson

Writing - Original Draft: Sushant Saluja, Danial Myers, and Simon G. Anderson

Supervision: Simon G. Anderson, Bernard D. Keavney, Freidoon Keshavarzi

Writing - Review & Editing: Simon G. Anderson, Bernard D. Keavney, Freidoon Keshavarzi

Project Administration: Simon G. Anderson, Bernard D. Keavney, Freidoon Keshavarzi

Validation: All authors

Conceptualisation: All authors

Special Thanks to Petra Jenkins, Vaikom S. Mahadevan, James McGowan, Heiko Schneider, Anna Dinsdale, Jaspal Dua, Bernard Clarke, and Andreas Hoschtitzky for their valuable proctoring assistance.

## Acknowledgement

Sushant Saluja is supported by the 4Ward North Wellcome Trust Clinical Research Training Fellowship (Grant Reference 203914/Z/16/Z). Simon G. Anderson was supported by an NIHR Academic Clinical Lectureship in Cardiology.
